# Potential actions for preventing high consumption of Non-Nutritive Sweeteners among Chilean children and adolescents: recommendations from a panel of relevant actors

**DOI:** 10.1017/S1368980024001745

**Published:** 2024-10-07

**Authors:** Marcela Reyes, Constanza Pino, Alejandra Ortega, Isabel Pemjean, Camila Corvalán, María Luisa Garmendia

**Affiliations:** 1 Institute of Nutrition and Food Technology (INTA), University of Chile, El Líbano 5524, Macul, Santiago, 7830490, Chile; 2 Doctoral Program in Public Health, School of Public Health, University of Chile, Santiago, Chile

**Keywords:** Non-nutritive sweeteners, Delphi technique, Dietary policies, Public health, Food formulation

## Abstract

**Objective:**

To provide local policymakers with a guideline of potential actions to prevent the high consumption of Non-Nutritive Sweeteners (NNS) among children and adolescents observed in Chile, given the potential health problems related to NNS intake.

**Design:**

The Delphi method was used for the evaluation of twenty-one recommendations to decrease the intake of NNS in paediatric population, with the participation of a panel of relevant actors.

**Setting:**

The proposed recommendations were developed by the research team using the NOURISHING framework; potential actions were based on the increase in the use and intake of NNS by Chilean children, current local food regulations, recommendations of health organisations and foreign policy experiences.

**Participants:**

Twenty-five relevant actors related to NNS, nutrition, food technology and paediatrics (out of thirty-nine invitations made to scholars, professional institutions and civil society’s organisations) participated in the Delphi study.

**Results:**

A consensus was reached on nine recommendations regarding relevance and feasibility to be part of the guideline. Recommendations involved measures mostly related to improving the delivery of information (food content and potential health effects of NNS), supporting the generation of more evidence of NNS health effects and substitutes, and marketing restrictions when targeted to children.

**Conclusions:**

The process produced a nine-action guideline to reduce the excessive NNS consumption among Chilean children and adolescents. Developed through a consensus-driven approach among key stakeholders, this guideline provides policymakers with a framework to adopt a precautionary stance, particularly concerning vulnerable populations, given the currently inconclusive evidence on the long-term health effects of NNS consumption.

## Graphical abstract

**Figure:**
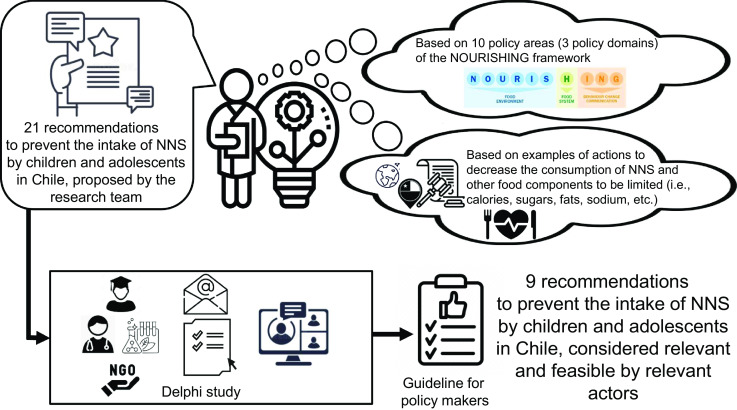

Non-nutritive sweeteners (NNS) have been encouraged as a replacement for added sugars. These additives are known for providing little to non-caloric content and having a greater sweet intensity than table sugar^(1)^. To guarantee the safety of its consumption, Acceptable Daily Intakes (ADI) have been established for each type of NNS (as well as for other food additives) by the Joint FAO/WHO Expert Committee on Food Additives (JECFA)^(2)^, and other institutions as the US Food and Drug Administration (FDA)^(3)^. ADI is the amount of a food additive, expressed on a body weight basis that can be ingested daily over a lifetime without appreciable health risk; it is typically based on toxicological studies^(4)^. However, recent observational studies have raised some concerns about the potentially harmful effects of NNS intake when consumed regularly under ADI limits. Negative effects on appetite regulation, glucose metabolism, microbiota composition, cardiovascular events, premature birth, among others, have been described particularly among pregnant women and infants^(5–8)^. Although results are still inconclusive^(9,10)^, some health associations such as the Institute of Medicine of the United States (IOM-USA) and the American Academy of Pediatrics have concluded that consumption of NNS in children should not be promoted until all its potential health effects are clarified^(11)^. Moreover, the WHO has launched a recommendation of avoiding the consumption of NNS for weight control among healthy population^(12)^. The recommendation is based on a recent systematic review and meta-analyses indicating that long-term studies associate higher intake of NNS with increased risk of obesity, type 2 diabetes and CVD^(13)^.

Despite the new concerns raised regarding the intake of NNS, those additives are broadly used in highly processed foods and beverages and -consequently highly consumed by the population where those products have an important dietary share. In Chile in 2017, 23 % of prepackaged food products contained at least one NNS, reaching 44 % among sweet prepackaged products^(14)^. The reported proportion greatly exceeds those available in Mexico (11 %), USA (4 %), Australia (< 1 %)^(15)^, New Zealand (5 %)^(16)^ and Hong Kong (4 %)^(17)^. In line with these results, studies on Chilean children showed that the vast majority of them (between 75 and 90 %) consumed foods or beverages with NNS on a daily basis^(18,19)^; these results are prior to the implementation of the Chilean Law on food labelling and food marketing, which mandates the use of warning labels and restricts sales and marketing for foods with high content of total sugars (as well as high content of energy, saturated fats and Na)^(20)^. The initial implementation of the regulation (which started in July 2016) was followed by an important decrease in the content of total sugars in packaged foods/beverages^(21)^ and a significant increase in the use of NNS (from 38 to 44 % among sweet products). Moreover, products decreasing the total sugars content were twelve times more likely to incorporate an NNS in their ingredient list^(14)^. In line with these results, the proportion of school-aged children consuming NNS the day before increased after the initial implementation of the warning labels, from 78 % in 2016 to 92 % in 2017^(22)^.

Chile has pioneered the use of front-of-package (FOP) warning labels and other structural measures for discouraging the consumption of foods considered unhealthy on the basis of their amount of energy and nutrients linked to non-communicable diseases (NCD)^(20)^. However, NNS use and intake have increased as an unintended consequence of this policy, which – in light of the emerging evidence – could imply a health concern. Thus, based on current local NNS intake, and global public policy experiences aimed at reducing NNS (or other food compounds when the former was non-available), we proposed a set of actions for local policymakers to consider in order to decrease the concerning intake of NNS among children in Chile. Those proposed actions were organised according to the three main policy domains systematised in the NOURISHING framework^(23)^, developed by the World Cancer Research Fund (WCRF): food environment, food system and behaviour change communication. Then, the set of actions was evaluated in terms of relevance and feasibility by a group of relevant actors, and the ones reaching consensus to be included in a recommendation guide were offered as such to policymakers. In this article, we report the methods and results of this experience. At the local level, this guide can help design and implement new measures aimed at addressing this unintended consequence of the food labelling regulation. On the other hand, we believe reporting this experience could be helpful in other contexts in the process of designing actions to prevent a high intake of NNS. Furthermore, this methodology could also be helpful as a starting point for food policy planning aimed to address different problems for which little experience (either local or foreign) is available. Developing a set of proposed actions organised in the NOURISHING framework and based on the closest policy experiences, which are later analysed and ranked according to relevance and feasibility by a panel of relevant actors, could be a useful basis for planning new action(s) to be implemented regarding a new food/ nutrition problem.

## Materials and methods

### Development of a set of proposed recommendations

Based on the three policy domains and ten policy areas systematised in the NOURISHING framework, we searched for different public policies aimed at reducing the intake of NNS or, when non-available, reducing the intake of other food components associated with NCD, as total sugars, saturated fats, *trans*-fats, Na, among others.

Actions targeting NNS were obtained mainly from those proposed by countries that have implemented (or are in the process of implementing) a warning label regulation, as the FOP precautionary legend for products that include sweeteners in Mexico^(24)^. We also looked for scientific papers and reports using search terms such as ‘non-nutritive sweeteners’ (or ‘non-caloric sweeteners’, ‘non-sugar sweeteners’ and ‘high-intensity sweeteners’) and food policies, as well as reviewing references used in different documents addressing the potential problem of NNS (i.e. guidelines and recommendations from different health or diet institutions). We complemented the search with other food policies when actions targeting specifically NNS were not found; for this purpose, we primarily reviewed the references organised at the NOURISHING website^(25)^ and also adapted local actions implemented for decreasing the intake of other food components that may be of concern^(26)^. Therefore, twenty-one recommendations to prevent excessive NNS intake in children and adolescents were formulated and organised according to the policy areas and food environment domains from the NOURISHING framework (Table 1). In some cases, little variations of a similar recommendation were offered (i.e. recommendations #1 and #2 or recommendations #4-6). Each recommendation was supported by a text indicating where the recommendation came from (i.e. developed by the research staff, from Mexican policy, etc.) and, when available, scientific articles or reports indicating the experience or evidence behind it (available in Spanish upon request).

**Table 1:** Initial set of proposed recommendations to prevent excessive Non-Nutritive Sweeteners (NNS) intake in Chilean children and adolescents, according to the NOURISHING framework and results of the evaluation of relevance and feasibility of rounds 1 and 2

Policy area	Recommendations by area	Relevant (R1)	Feasible (R1)	Relevant (R2)	Feasible (R2)
Nutrition label standards and regulations on the use of claims and implied claims on food	1. To inform the presence of NNS on the front of the package of foods and beverages: *via warning message (i.e. stop sign).*	88	82	Not evaluated (there is an option with better performance)	Not evaluated (there is an option with better performance)
	2. To inform the presence of NNS on the front of the package of foods and beverages: *via precautionary legend (e.g. as the messages used in Mexico).*	94	82	Not evaluated (already agreed to be included)	Not evaluated (already agreed to be included)
	3. To ban the use of nutritional or health declarations in food and beverages containing NNS.	71	71	60	65
Offer healthy food and set standards in public institutions and other specific settings	4. To ban the sale of food and beverages containing NNS in school environments (e.g. kiosks, school cafeteria, coffee shop and food programmes): *only at kindergartens or preschools.*	71	71	65	75
	5. To ban the sale of food and beverages containing NNS in school environments (e.g. kiosks, school cafeterias and food programmes): *only at schools.*	65	65	Not evaluated (there is an option with better performance)	Not evaluated (there is an option with better performance)
	6. To ban the sale of food and beverages containing NNS in school environments (e.g. kiosks, coffee shops and food programmes): *at schools and preschools.*	71	65	Not evaluated (there is an option with better performance)	Not evaluated (there is an option with better performance)
Use economic tools to address food affordability and purchase incentives	7. To implement taxations for food and beverages containing NNS.	59	24	20	35
	8. To ban special offers (e.g. reduced cost, 2 × 1) for food and beverages containing NNS.	59	59	50	55
Restrict food advertising and other forms of commercial promotion	9. To restrict marketing directed to children for food and beverages containing NNS.	94	82	Not evaluated (already agreed to be included)	Not evaluated (already agreed to be included)
Improve nutritional quality of the whole food supply	10. To regulate NNS content in packaged foods and beverages: *banning the use of NNS.*	35	18	Not evaluated (already agreed to be discarded)	Not evaluated (already agreed to be discarded)
	11. To regulate NNS content in packaged foods and beverages: *establishing an allowed NNS maximum concentration.*	88	71	80	50
	12. To regulate NNS content in packaged foods and beverages: *establishing a maximum number of NNS types allowed to be used in each product.*	71	65	70	65
	13. To enable and/or strengthen the existing control over the veracity of NNS labelling with a risk-based approach (focus on the main foods/beverages sources of NNS).	88	77	Not evaluated (already agreed to be included)	Not evaluated (already agreed to be included)
Set incentives and rules to create a healthy retail and food service environment	14. To ban the sale of food and beverages containing NNS within 100 m of schools and preschools.	35	47	Not evaluated (already agreed to be discarded)	Not evaluated (already agreed to be discarded)
	15. To ban positioning of foods and beverages containing NNS in privileged shelve areas in stores (i.e. those more visible to children and those in checkout lines).	82	82	Not evaluated (already agreed to be included)	Not evaluated (already agreed to be included)
Harness food supply chain and actions across sectors to ensure coherence with health	16. To promote research and innovation* focused on NNS: monitoring the presence in foods and beverages; identifying technological alternatives for its reduction or replacement; monitoring intake in at-risk populations; studying short-, medium- and long-term health effects. Enabling special calls or priorities in existing grants (i.e. FONDEF and FONIS in Chile, and others).^*^	94	76	Not evaluated (already agreed to be included)	Not evaluated (already agreed to be included)
Inform people about food and nutrition through public awareness	17. To implement a health campaign on social media, radio and television to promote healthy eating and discourage the intake of NNS in children and adolescents.	82	88	Not evaluated (already agreed to be included)	Not evaluated (already agreed to be included)
	18. To add warning messages to discourage the intake of NNS in Food-Based Dietary Guidelines directed to general population.^*^	76	71	90	75
Nutrition advice and counselling in healthcare settings	19. To include (or periodically update, if applicable) warning messages to discourage the intake of NNS in Clinical Guidelines for specific subpopulations such as children under 2 years old, paediatric population, or pregnant and breast-feeding women.	94	88	Not evaluated (already agreed to be included)	Not evaluated (already agreed to be included)
Give nutrition education and skills	20. To include in school curriculum topics such as the health effects of NNS and cooking classes (e.g. decreasing the sweetness of preparations).	71	65	60	55
	21. To include the potential health effects of NNS in the curriculum of university programmes focused on health (Nutrition, Nursery, and Medicine, among others).	82	76	Not evaluated (already agreed to be included)	Not evaluated (already agreed to be included)

The NOURISHING framework’s domains are indicated with colours. N-O-U-R-I-S: recommendations of the Food Environment domain; H: recommendations of the Food System domain; I-N-G: recommendations of the Behavior Change Communication domain. Recommendations #1 and #2 are variations of the action ‘To inform the presence of NNS in the front of the package of foods and beverages’. Recommendations #4, #5, and #6 are variations of the action ‘To ban the sale of food and beverages containing NNS in school environments’. Recommendations #10, #11 and #12 are variations of the action ‘To regulate NNS content in packaged foods and beverages’.

Values of the right columns represent the proportion of panellist considering a specific recommendation relevant + very relevant, or feasible + very feasible (R1), and relevant or feasible (R2).

Recommendations reached a consensus to be included in the guideline if => 75 % of panellists considered them ‘relevant’ and ‘feasible’. They reached a consensus to be discarded from the guideline if < 50 % of panellists considered them ‘relevant’ or ‘feasible’. In R1, for recommendations that were variations of the same actions, the recommendation with best performance was selected.

* For the second round of evaluation (R2), recommendation #18 was reformulated as follows: ‘To add warning messages to discourage the intake of NNS in Food-Based Dietary Guidelines orientated to populations potentially at risk, like pregnant women and children’.

### Process of evaluation of recommendations

The evaluation process was carried out using a two-round Delphi method and a synchronous workshop with the participation of scholars, *ad hoc* professionals (i.e. working on diet, health or food technology), consumers’ associations and public health experts from different institutions. Delphi is an evaluation method that consists of a communication process between experts organised in a group panel to reach an agreement through systematically recollecting judgements about a topic in order to process this information and build a consensus^(27)^. The process was carried out in almost 7 weeks (between September and November 2021), using two questionnaires available at REDCAP^(28)^ and an online synchronous workshop via Zoom to review the questionnaire’s results and verify the agreements.

#### Conformation of the panel of relevant actors

To build a pertinent and diverse panel, the participation of relevant actors from academic institutions and civil society was considered. Food industry representatives were not included in the panel because it was considered they would have strong conflicts of interest.

For the identification of academic scholars, a bibliographic review was made to identify local researchers who have authored or co-authored at least one article about NNS use or consumption in Chilean populations and public policies associated with food and nutrition. From this review, twenty-one relevant actors were invited to participate in the expert panel; sixteen accepted the invitation, nine completed the first questionnaire, ten completed the second questionnaire and eleven participated in the synchronic workshop (see online supplementary material, Supplemental Table 1 and Supplemental Figure 1).

Professionals and civil societies whose objectives related to the themes of NNS consumption, nutrition, food technology and paediatrics were invited to participate. The research team identified eighteen active societies and food research centres; eleven accepted the invitation (four civil organisations, three professional societies and three food research centres). All organisations were asked to select the most suitable representative to participate in the Delphi panel; therefore, no specific background or time of experience was asked (see online supplementary material, Supplemental Table 2). Eight societies and food research centres representatives completed the first questionnaire, ten completed the second questionnaire and seven participated in the synchronic workshop. In total, twenty-five relevant actors participated in at least one of the three evaluation stages (see online supplementary material, Supplemental Figure 1).

The invitations to participate were delivered via email, including a summary of the project’s results and a description of the process for evaluating the recommendations. All participants gave their Informed Consent and signed the Confidentiality Agreement and Conflict of Interests Declaration documents.

#### Evaluation of the recommendations

To evaluate the recommendations, we conducted two rounds with online questionnaires (using REDCAP). Participants could access the questionnaires from any electronic device (laptop, tablet and cellphone) with an URL and could save their progress to continue at any moment.

Relevant actors were asked to rate each of the twenty-one recommendations regarding relevance and feasibility using four-level Likert questions (4 = very relevant/feasible, 1 = non-relevant/feasible) and include a brief justification of their answers. Relevance was defined as the importance of the recommendation in preventing excessive NNS consumption, and feasibility was defined as the plausibility of implementing this recommendation in Chile, considering available resources, technical capabilities and possible application in the next 3–5 years. All recommendations included a link to a supplementary document with the background information used to formulate each recommendation and six questions about the clarity, pertinence of the language and the additional information of each recommendation (see online supplementary material, Supplemental Table 3 shows details about the structure of the first questionnaire). At the end of the first questionnaire, there was a final section with open questions asking participants to suggest further recommendations for preventing a high intake of NNS by children and adolescents. Participants were also encouraged to provide any additional comments regarding the proposed recommendations. Questionnaires were piloted between five team members of CIAPEC-INTA not associated with the current study for clearness and comprehension before being used with relevant actors.

A second questionnaire was sent 2 weeks after the first one, displaying the recommendations that did not reach consensus (either to be included or to be excluded of the guideline) on the first round. In this round, participants were asked to rate whether the recommendations were relevant or feasible using dichotomous answers (yes/no). To inform their decision, experts were provided with the first-round results (including the main aspects detected from justifications) and the links to the supplementary information documents. All relevant actors mapped were invited to participate in the second questionnaire, even if they did not answer the first one (see online supplementary material, Supplemental Table 4 shows more details about the structure of the second questionnaire).

Finally, a synchronous workshop was held online to rank the recommendations that reached consensus. A summary of prior rounds of Delphi study were presented, highlighting the selected recommendations. Participants were invited to select – in first place – the three most relevant recommendations, then a moderately relevant pack of three recommendations and finally a set of three recommendations with the lowest relevance. The same exercise was done to rank feasibility. The ranking exercises were done in REDCAP platform; results were informed during the workshop, and then discussion was encouraged in order participants could make open comments about the workshop’s results and their own ranking options. Participants were not invited to change their responses already submitted if wanted. All the mapped scholars, professionals, civil societies and researchers from food research centres were invited, even if they did not participate in previous rounds.

#### Analysis

For the first questionnaire, consensus criteria were established by clustering the Likert options into categories: ‘relevant’ (options 3 and 4 for relevancy), ‘non-relevant’ (options 1 and 2 for relevancy), ‘feasible’ (options 3 and 4 for feasibility) and ‘non-feasible’ (options 1 and 2 for feasibility).

In each round, the twenty-one recommendations were classified into three consensus categories:

- Consensus to be included in the guideline: those considered ‘relevant’ and ‘feasible’ by => 75 % of panellists.
- Consensus to be excluded from the guideline: those considered ‘relevant’ or ‘feasible’ by < 50 % of panellists.
- No consensus: those considered ‘relevant’ or ‘feasible’ by 50–75 % of panellists.

We also conducted a qualitative content analysis of the answers provided to the open questions. Using the Grounded Theory approach, we identified the arguments that were most frequently used to justify the relevance and feasibility of each recommendation; we also conducted an axial analysis to further explain the panellists’ standpoint. All analyses were performed using the ATLAS.ti Scientific Software Development GmbH, version 7.5.18, Licensed by Cincom Systems, Inc.

For the second questionnaire, the same three consensus categories were computed; given in this round just dichotomous responses (i.e. relevant/feasible *v*. non-relevant/feasible) were available, no further clustering of responses was needed. Finally, for the ranking exercise, an agreement score was calculated based on how many times a recommendation was categorised in each of the three levels for relevance and feasibility (most relevant/feasible = 1, medium relevant/feasible = 2 and less relevant/feasible = 3). A total score was obtained by adding the relevance and feasibility scores for each recommendation and used to rank the recommendations from the lowest (higher relevance/feasibility) to the highest score.

## Results

### Selection of recommendations to be included in the guideline

Overall, more than 80 % of the panellists thought the twenty-one recommendations were clear and used pertinent language for its target population. Evidence provided to judge the recommendations was also considered sufficient, except for recommendations #7 and #10, where more than half of the panel considered these recommendations did not have enough supporting information (see online supplementary material, Supplemental Table 5).

The results of the evaluation of relevance and feasibility are presented in Table 1. In the first round, nine out of twenty-one recommendations reached consensus to be included in the guideline (i.e. at least 75 % of panellists considered them to be both relevant and feasible), mainly those related to labelling (#1, #2), restricting marketing (#9), monitoring NNS content of foods (#13), banning placement strategies (#15), promoting food innovation (#16), informing consumers through campaigns, schools or packages (#17, #19 and #21). Since recommendations #1 and #2 were variations of the same action, we selected only #2 to be included in the guideline, considering it got a higher score in relevancy than recommendation #1. Two recommendations reached a consensus to be excluded from the guideline (i.e. 50 % or less of panellists considered them to be both relevant and feasible: banning NNS use (#10) and banning selling foods with NNS close to schools (#14).

Ten recommendations did not reach consensus (i.e. were considered ‘relevant’ or ‘feasible’ by 50–75 % of panellists): #3, #4, #5, #6, #7, #8, #11, #12, #18 and #20; those had to be re-evaluated in the second round. Recommendations #4, #5 and #6 were just one recommendation with little variation among them (i.e. institutions to be included in the restriction); thus, we only retained #4 because it had the highest feasibility and relevance scores. Therefore, in the second round, eight recommendations were evaluated; only recommendation #18 had consensus to be included in the guideline (after revising the wording, see methods section), and recommendation #7 had consensus to be excluded in the guideline (taxation of foods with NNS). The remaining six recommendations got over 50 % in relevance or feasibility but below 75 %, meaning they did not reach a consensus. With these results, the evaluation panel agreed on nine recommendations to be included in the guide. These recommendations encompassed four from the Food Environment domain (#2, #9, #13 and #15), four from the Behavior Change Communication domain (#17, #18, #19 and #21), and one from the Food System domain (#16), as stipulated by the NOURISHING framework.

### Content analysis results

During the first round of Delphi, experts were asked to write down justifications for their responses. The content analysis of these responses revealed four emerging categories to classify the recommendations: (i) making structural changes to improve information transparency, (ii) making structural changes aimed to prohibit or limit the offer of NNS-containing products, (iii) generating new evidence about NNS and (iv) strengthening health communication (see online supplementary material, Supplemental Table 6).

The structural recommendations focused on transparency of information (i.e. FOP information on the use of NNS and strengthening the control of veracity of the labels) may have reached a consensus given their relevance to include straightforward information about the food’s NNS content. Also, pragmatism could have contributed since there are similar regulations previously implemented in Chile^(20)^. However, more panellists preferred a precautionary legend than a warning label, which was justified by the lack of enough evidence about the potential damage of NNS to health.

The panel was also against most structural changes aimed to prohibit or limit the offer of NNS-containing products (i.e. modulate the offer (O) or banning/limiting the use of NNS on foods/beverages (I)): *‘There is also no clear evidence that they are harmful to children, and eventually, they could help, along with other measures, to reduce sugar consumption’* (Scholar, ID No. 3); *‘I don’t believe it’s necessary to prohibit the consumption of these ingredients. There are others that are more harmful, such as alcohol, and there is no consumption prohibition.’* (Scholar, ID No. 4). In fact, some experts commented that actions like this could be counterproductive because people could increase sugars consumption if NNS were not available and because this could limit alternatives for some specific groups, such as diabetics and children with obesity. Only regulations related to limiting marketing of NNS targeted to children were approved because the panel believed a precautionary principle should be followed in this age group given the uncertainty of the evidence. Those actions were already in place for foods with high content of energy or nutrients linked to NCD. Finally, some recommendations were considered unfeasible since the ADI limits already exist, and there is not enough information to justify using different safety limits.

The generation of new evidence around NNS was supported by the fact that most panellists considered that there was a lack of solid evidence of the potential health effects of different levels of NNS, particularly among children. Therefore, panellists considered these aspects need urgent clarification to support policy actions.

About the emerging category of strengthening health communication, only recommendation #20 did not reach a consensus to be added to the guide because of the lack of technical and infrastructural feasibility to be implemented in schools. Most communication recommendations reached a consensus regarding relevance and feasibility and were directed to the general public and at-risk population groups. Panellists found that the local Food-Based Dietary Guidelines are already periodically updated, which makes it easier to add information about NNS: *‘These guidelines must be reviewed constantly. Thus, adding a recommendation or updating them seems absolutely feasible’* (Scholar, ID No. 26). Similarly, the curricula of university programmes are regularly updated as well, and they could help health professionals to learn more about the effects of NNS. Above all, health promotion campaigns were considered a cost-effective and easy-to-implement measure that must be coordinated alongside other actions: *‘In order to achieve the objective of all the previous recommendations, it is essential to have an educational campaign in the media, since the population, in general, uses this type of venues to gain information’* (Civil Society Representative, ID No. 10).

General comments regarding proposed recommendations or promotion healthier diets were done; however, no new recommendations targeted to prevent the excessive intake of NNS by children and adolescents were proposed by the evaluation panel members.

### Ranking of recommendations

The total scores for each recommendation and their prioritisation ranking are shown in Table 2. Recommendation #2, ‘To inform the presence of NNS on the front of the package of foods and beverages: via precautionary legend (e.g. as the messages used in Mexico)’, obtained the highest priority, followed by recommendation #9, ‘To restrict marketing directed to children for food and beverages containing NNS’, both of them from the Food Environment domain of the NOURISHING framework. The remaining seven recommendations got somewhat similar scores, being the lowest priority for the recommendation ‘To include the potential health effects of NNS in the curriculum of university programmes focused on health (Nutrition, Nursery, and Medicine, among others)’.

**Table 2:** Ranking of recommendations to prevent excessive Non-Nutritive Sweeteners (NNS) intake in Chilean children and adolescents by relevance, feasibility and total score

Ranking	Recommendation	Relevance score	Feasibility score	Total score
1	#2. To inform the presence of NNS on the front of the package of foods and beverages: via precautionary legend (e.g. as the messages used in Mexico).	20	26	46
2	#9. To restrict marketing directed to children for food and beverages containing NNS.	29	33	62
3	#16. To promote research and innovation focused on NNS: monitoring the presence in foods and beverages; identifying technological alternatives for its reduction or replacement; monitoring intake in at-risk populations; studying short-, medium- and long-term health effects. #13. To enable and/or strengthen the existing control over the veracity of NNS labelling with a risk-based approach (focus on the main foods/beverages sources of NNS).	34	39	73
4	#17. To implement a health campaign on social media, radio and television to promote healthy eating and discourage the intake of NNS in children and adolescents.	38	36	74
5	#19. To include (or periodically update, if applicable) warning messages to discourage the intake of NNS in Clinical Guidelines for specific subpopulations such as children under 2 years old, paediatric population, or pregnant and breast-feeding women.	41	35	76
6	#18. To add warning messages to discourage the intake of NNS in Food-Based Dietary Guidelines orientated to populations potentially at risk, like pregnant women and children.	43	37	80
7	#15. To ban positioning of foods and beverages containing NNS in privileged shelve areas in stores (i.e. those more visible to children and those in checkout lines).	43	38	81
8	#21. To include the potential health effects of NNS in the curriculum of university programmes focused on health (Nutrition, Nursery, and Medicine, among others).	42	41	83

Relevance/feasibility scores were computed for each recommendation by adding the values assigned to each level of relevance or feasibility (most relevant/feasible = 1, medium relevant/feasible = 2, less relevant/feasible = 3). A total score was obtained by adding the relevance and feasibility scores. Given fourteen persons participated in the final workshop, total scores could range from 28 to 84. Recommendations were ranked from the lowest (higher relevance/feasibility) to the highest score.

## Discussion

This study aimed to assess a set of recommendations to prevent the excessive consumption of NNS in children and adolescents. Using the Delphi method, nine out of twenty-one proposed recommendations reached a consensus to be included in a guide to be presented to policymakers. The evaluation panel prioritised recommendations oriented to (1) provide reliable information to consumers on food package labels, (2) restrict marketing to children for products containing NNS, (3) promote research about NNS and (4) communication to change behaviours regarding NNS intake.

Recently, WHO launched a new guideline for NNS (or non-sugar sweeteners, according to WHO), which recommends against the use of these additives to control body weight or reduce the risk of NCD^(12)^. Previously, the WHO regional offices for Europe and the Americas had considered in their nutrient profiles the presence of NNS as criteria for discouraging the consumption of some food products. In 2015, the Europe Office restricted the marketing to children among beverages containing NNS (i.e. dairy beverages, drinks with almond, soya, rice and oatmeal, soft drinks, lemonade, orange drinks, mineral or flavoured water); this was recently updated to a threshold of 0 g/100 g for NNS for all food categories, both foods and beverages^(29)^. In 2016, the Pan American Health Organization indicated that processed and ultra-processed foods (UPF) containing NNS or high levels of nutrients linked with NCD may be subject to various food environment regulations. These regulations include warning labels, price adjustments, marketing restrictions and prohibitions on inclusion in school foods environment, among others. These measures would differentiate them from processed and UPF that do not contain NNS or high levels of nutrients associated with NCD^(30)^. Moreover, some international agencies have provided explicit recommendations regarding NNS intake among children^(11)^. The American Academy of Pediatrics and the Ministry of Health of Brazil recommend avoiding NNS consumption in children because of the lack of evidence about their effects^(31,32)^. Similarly, the Agriculture Department and the 2020–2025 dietary guidelines of the USA and the Ministry of Health of Uruguay recommend avoiding NNS consumption during the first 2–3 years of age^(33,34)^. However, other governmental agencies like the FDA and scientific organisations such as the Mexican Society of Pediatrics and the Latin-American Association of Diabetes suggest that NNS consumption in children is healthy within ADI limits^(11,35,36)^.

Even when several health/diet organisations suggest NNS intake should be avoided, at least among young children, only a few countries have implemented specific regulations to prevent the excessive consumption of NNS. Mexico recently implemented an FOP precautionary legend to declare the presence of NNS in packaged foods^(37)^. Similarly, regulation No 1169/2011 of the European Parliament establishes that food or beverages with NNS or a combination of these additives should have the message ‘contains sweeteners’ near the product’s name^(38)^. In Canada, it is mandatory to show a declaration when the product contains aspartame, sucralose, acesulfame-k or neotame^(39)^. South Africa and Colombia are discussing food labelling regulations that would include warning labels for foods containing NNS^(40,41)^. We are unaware of other policies aimed at halting the increase of NNS consumption among children; however, a systematic multicounty comparison of policies targeting NNS is beyond the scope of this study. Given the new WHO recommendation and the call for increasing research regarding the safety of chronic exposure to NNS within ADI, this will likely become a very dynamic scenario.

In our study, the selected recommendations correspond to the three domains of the NOURISHING framework: Food Environment (*n* 4), Behavior Change Communication (*n* 4) and Food System (*n* 1). The two recommendations that reached the first and second places in the ranking belonged to the Food Environment domain. It should be noted that these two recommendations are very similar to other regulations already implemented in Chile that have shown positive results. Since implementing the Law of Food Labelling and Advertising in Chile, there has been a significant decrease in the purchase of food with warning FOP labels^(42,43)^. Similarly, after the marketing regulation imposed by this Law, there was a 35 % decrease in the television spots of unhealthy foods/beverages targeted to preschoolers and a 52 % decrease in the spots targeted to adolescents^(44)^. These positive results likely influenced panellists to suggest similar kinds of regulations to discourage the consumption of NNS in children.

Regarding the Behavior Change Communication domain, only one of the recommendations did not reach a consensus to be included in the guideline. Experts recognise the importance and feasibility of actions related to improving information to prevent excessive NNS consumption. Given that evidence is still inconclusive about the adverse effects of NNS^(31,32)^, the panel felt it was safer to improve people’s information rather than implement structural actions to decrease consumption. Actually, even when the most prioritised recommendation is part of the Food Environment domain, it is also targeted to increase population awareness by displaying easy-to-see and easy-to-understand information. Evidence suggests that massive health campaigns could have mixed effects depending of the application and the target behaviour^(45,46)^, and the impact of including warning messages in Dietary Guidelines, particularly for pregnant and lactating women and infants, would likely depend on the influence that those guidelines have among healthcare visits and food programmes, or food policy^(3)^. Chile has recently revised its Food-Based Dietary Guidelines, including a recommendation to avoid UPF and foods with a warning label FOP for the first time but did not include a specific message regarding NNS^(47)^. Regarding the Food System domain, the promotion of local research and innovation linked to NNS is in line with the new NNS WHO guideline recommendation, which makes a clear call to provide further evidence of the health impact of long-term exposure to NNS^(12)^.

Several recommendations proposed by the research staff reached a consensus to be excluded of the guideline. According to the relevant actors’ comments, these actions seemed too restrictive, considering there is non-conclusive evidence regarding the adverse health effects of NNS intake. The Chilean food regulation does not consider substantial restrictions (as recommendations #7 and #10) to other food components, which current intake has been considered a health risk factor, such as sugars and Na. Panellists’ considerations may change in the future if new evidence on the health effects of NNS exposure emerges, local regulations to ‘high in’ foods expand to include price regulations or other actions aimed at shaping healthier food environments. Evidence suggests that structural actions that facilitate healthier dietary decisions would be more cost-effective than focusing on individual behavioural changes^(48)^; considering these recommendations could lead to more effective food policy planning.

Although various recommendations reached a consensus after this iterative process with a heterogeneous relevant actors’ panel, some aspects that could work against the evaluation should be considered. First, it is likely that a different research team could have developed a different set of recommendations (at least regarding the framing of them), even by following the same methodology. Thus, the set of twenty-one recommendations evaluated in the first round of the panel may be influenced by the pre-existent ideas and the experiences of researchers leading the study; using the NOURISHING framework to develop *ad hoc* recommendations in every policy area should have decreased the extent in which personal opinions shaped the recommendations. Second, the assessment of twenty-one recommendations simultaneously could be an exhaustive process for the participants; this was considered for the second round, which required less time to be completed. Another limitation is that the open questions with explanations for relevance and feasibility were only included in the first round, so it is possible that we could have lost some of the panel’s opinions; however, panellists also had the possibility of expressing their opinions in the synchronous workshop. On the other hand, in order to encourage the participation of most actors identified as relevant for the Delphi process, all of them were invited to respond to both questionnaires and to participate in the workshop, even if they were not part of the prior round(s). This might have limited the ability to reach consensus, but at the same time, it provides robustness to the agreements reached. Finally, other methods for arriving at consensus have been described with adequate performance, such as the nominal group technique^(49,50)^; however, the Delphi approach has been applied by our and other teams successfully as part of the Food-Epi component of the INFORMAS network^(51)^.

At the end of the process, the set of nine recommendations agreed upon by the relevant actors’ panel was compiled in a guideline presented to *ad hoc* authorities of the Ministry of Health (and re-presented when authorities changed). The recommendations were contextualised with results on the use of NNS in foods and beverages, the intake of those additives among the paediatric population, a local legal mapping and the methodology used to arrive to the set of recommendations.

### Conclusion

Nine potential actions for preventing a high intake of NNS among children and adolescents from Chile were set as a recommendation guide for health authorities. These recommendations were developed based on what different health organisations suggested and foreign experiences aimed at avoiding NNS intake (when available) or by experiences discouraging the intake of other food components considered unhealthy. The recommendations were organised in different policy areas and domains, based on the NOURISHING framework, and evaluated by a heterogeneous expert panel that reached a consensus on the actions that should be included in the guideline. The participation of experts from a wide range of local organisations may act, on the one hand, as an endorsement for policymakers that would like to use these recommendations to inform policy planning and, on the other hand, to increase the options to obtain policy support from relevant actors from academia, professional and civic organisations. We believe this guideline constitutes a useful tool for policymakers to advance on protective policies regarding health/nutrition topics in which evidence is still inconclusive (or new controversial evidence appears). Based on the precautionary principle, it seems sound to prevent the high intake of a food component when evidence of safety to its chronic exposure is not consistent, at least for populations at vulnerable life periods such as children, adolescents and pregnant women.

## Supporting information

Reyes et al. supplementary material Reyes et al. supplementary material

## Supplementary material

For supplementary material accompanying this paper visit https://doi.org/10.1017/S1368980024001745

## References

[1] Das A & Chakraborty R (2018) An Introduction to Sweeteners. London: Elsevier. pp. 1–13.

[2] FAO Joint FAO/WHO Expert Committee on Food Additives (JECFA) (2024) https://www.who.int/groups/joint-fao-who-expert-committee-on-food-additives-(jecfa) (accessed June 2024).

[3] FDA (2017) High-Intensity Sweeteners. https://www.fda.gov/food/food-additives-petitions/high-intensity-sweeteners (accessed June 2024).

[4] FAO & WHO Codex Alimentarius (1997) General Standard for Food Additives. https://www.fao.org/4/ac335e/ac335e04.htm#:~:text=Acceptable%20Daily%20Intake%20(ADI)%20is,standard%20man%20%3D%2060%20kg) (accessed on June 2024).

[5] Suez J , Korem T , Zeevi D et al. (2014) Artificial sweeteners induce glucose intolerance by altering the gut microbiota. Nature 514, 181–186. 25231862 10.1038/nature13793

[6] Gupta G , Yograj S , Gupta AK et al. (2017) Relationship between intake of artificial sweeteners and body mass index in young non-diabetic adults: a cross-sectional study. Int J Res Med Sci 5, 1208–1212.

[7] Gardener H , Rundek T , Markert M et al. (2012) Diet soft drink consumption is associated with an increased risk of vascular events in the Northern Manhattan Study. J Gen Intern Med 27, 1120–1126. 22282311 10.1007/s11606-011-1968-2 PMC3514985

[8] Halldorsson TI , Strøm M , Petersen SB et al. (2010) Intake of artificially sweetened soft drinks and risk of preterm delivery: a prospective cohort study in 59 334 Danish pregnant women. Am J Clin Nutr 92, 626–633. 20592133 10.3945/ajcn.2009.28968

[9] Laviada-Molina H , Molina-Segui F , Pérez-Gaxiola G et al. (2020) Effects of nonnutritive sweeteners on body weight and BMI in diverse clinical contexts: systematic review and meta-analysis. Obes Rev 21, e13020. 32216045 10.1111/obr.13020

[10] Rogers PJ & Appleton KM (2021) The effects of low-calorie sweeteners on energy intake and body weight: a systematic review and meta-analyses of sustained intervention studies. Int J Obes (Lond) 45, 464–478. 33168917 10.1038/s41366-020-00704-2

[11] Sylvetsky A , Rother KI & Brown R (2011) Artificial sweetener use among children: epidemiology, recommendations, metabolic outcomes, and future directions. Pediatr Clin North Am 58, 1467–1480. 22093863 10.1016/j.pcl.2011.09.007 PMC3220878

[12] World Health Organization (2023) Use of Non-Sugar Sweeteners: WHO Guideline. Geneva: World Health Organization. 37256996

[13] World Health Organization, Rios-Leyvraz M & Montez J (2022) Health Effects of the Use of Non-Sugar Sweeteners: A Systematic Review and Meta-Analysis. Geneva: World Health Organization.

[14] Zancheta C , Corvalán C , Smith Taillie L et al. (2021) Changes in the use of non-nutritive sweeteners in the Chilean food and beverage supply after the implementation of the food labeling and advertising law. Front Nutr 8, 773450. 34859036 10.3389/fnut.2021.773450 PMC8630583

[15] Dunford EK , Taillie LS , Miles DR et al. (2018) Non-nutritive sweeteners in the packaged food supply—an assessment across 4 countries. Nutrients 10, 257. 29495259 10.3390/nu10020257 PMC5852833

[16] Nunn R , Young L & Mhurchu CN (2021) Prevalence and types of non-nutritive sweeteners in the New Zealand Food Supply, 2013 and 2019. Nutrients 13, 3228. 34579101 10.3390/nu13093228 PMC8471995

[17] Coyle DH , Dunford EK , Wu JH et al. (2021) The use of non-nutritive and low-calorie sweeteners in 19 915 local and imported pre-packaged foods in Hong Kong. Nutrients 13, 1861. 34072564 10.3390/nu13061861 PMC8229473

[18] Duran S , Oñate G & Haro P (2014) Consumption of non-nutritive sweeteners and nutritional status in 10–16 year old students. Arch Argent Pediatr 112, 207–214. 24862801 10.5546/aap.2014.eng.207

[19] Venegas Hargous C , Reyes M , Smith Taillie L et al. (2020) Consumption of non-nutritive sweeteners by pre-schoolers of the Food and Environment Chilean Cohort (FECHIC) before the implementation of the Chilean Food Labelling and Advertising Law. Nutr J 19, 69. 32650775 10.1186/s12937-020-00583-3 PMC7353755

[20] Corvalan C , Reyes M , Garmendia ML et al. (2019) Structural responses to the obesity and non-communicable diseases epidemic: update on the Chilean Law of Food Labelling and Advertising. Obes Rev 20, 367–374. 30549191 10.1111/obr.12802

[21] Reyes M , Smith Taillie L , Popkin B et al. (2020) Changes in the amount of nutrient of packaged foods and beverages after the initial implementation of the Chilean Law of Food Labelling and Advertising: a nonexperimental prospective study. PLoS Med 17, e1003220-e. 32722710 10.1371/journal.pmed.1003220 PMC7386631

[22] Rebolledo N , Reyes M , Popkin B et al. (2022) Changes in nonnutritive sweetener intake in a cohort of preschoolers after the implementation of Chile’s Law of Food Labeling and Advertising. Pediatr Obes 17, e12895. 35088571 10.1111/ijpo.12895

[23] Hawkes C , Jewell J & Allen K (2013) A food policy package for healthy diets and the prevention of obesity and diet-related non-communicable diseases: the NOURISHING framework. Obes Rev 2, 159–168. 10.1111/obr.12098 24103073

[24] Ministry of Economy (2020) Modification to the Mexican Official Standard NOM-051-SCIFI/SSA1-2010. Official Gazette. 1:1. https://www.dof.gob.mx/2020/SEECO/NOM_051.pdf (accesed June 2024).

[25] World Cancer Research Fund International (2015) Our Policy Framework to Promote Healthy Diets & Reduce Obesity. https://www.wcrf.org/int/policy/nourishing/our-policy-framework-promote-healthy-diets-reduce-obesity (accessed June 2021).

[26] Rodríguez-Osiac L , Fernandes ACP , Mujica-Coopman MF et al. (2021) A description of Chilean food and nutrition health policies. Rev Med Chil 149, 1485–1494. 35319638 10.4067/s0034-98872021001001485

[27] González Muñoz Y , Palomino C , Pérez E et al. (2018) Applications and future trends of expert consultation in the food sector: an overview of the Delphi methodology. Actualización en Nutrición 19, 55–68.

[28] Harris PA , Taylor R , Thielke R et al. (2009) Research electronic data capture (REDCap)--a metadata-driven methodology and workflow process for providing translational research informatics support. J Biomed Inform 42, 377–381. 18929686 10.1016/j.jbi.2008.08.010 PMC2700030

[29] WHO Regional Office for Europe (2023) WHO Regional Office for Europe: Nutrient Profile Model. Report No.: 2023-6894-46660-68492. Copenhagen, Denmark: WHO Regional Office for Europe.

[30] Pan American Health Organization (PAHO) (2016) Pan American Health Organization Nutrient Profile Model. Washington, DC: PAHO.

[31] Baker-Smith CM , de Ferranti SD , Cochran WJ et al. (2019) The use of nonnutritive sweeteners in children. Pediatrics 144, e20192765. 31659005 10.1542/peds.2019-2765

[32] Ministério da Saúde do Brasil (2019) Dietary Guidelines for Brazilian Children under Two Years of Age. Brasília, Brazil: Ministério da Saúde do Brasil. p. 265.

[33] U.S. Department of Agriculture & U.S. Department of Health and Human Services (2020) Dietary Guidelines for Americans, 2020–2025, 9th ed. Washington, DC: USDA, USDHHS.

[34] Ministry of Public Health of Uruguay (2017) Complementary feeding guide for children between 6 and 24 months. https://www.gub.uy/ministerio-desarrollo-social/comunicacion/publicaciones/guia-alimentacion-complementaria-para-ninos-entre-6-24-meses (accessed June 2024).

[35] Laviada-Molina H , Escobar-Duque ID , Pereyra E et al. (2018) Consensus of the Latin American Diabetes Association on the use of non-caloric sweeteners in people with diabetes. ALAD 8, 152–174.

[36] Wakida-Kuzunoki GH , Aguiñaga-Villaseñor RG , Avilés-Cobián R et al. (2017) Consensus of the Latin American Diabetes Association on the use of non-caloric sweeteners in people with diabetes. Rev Mex Pediatr 84, S3–S23.

[37] White M & Barquera S (2020) Mexico adopts food warning labels, why now? Health Syst Reform 6, e1752063. 32486930 10.1080/23288604.2020.1752063

[38] European Parliament and the Council (2011) Regulation No 1169/2011. =https://eur-lex.europa.eu/LexUriServ/LexUriServ.do?uri=OJ:L:2011:304:0018:0063:en:PDF (accessed June 2024).

[39] Government of Canada Labelling requirements for sweeteners and foods that contain sweeteners - Food label requirements - Canadian Food Inspection Agency (2024) https://inspection.canada.ca/en/food-labels/labelling/industry/sweeteners (accessed June 2024).

[40] Ministry of Health and Social Protection of Colombia (2023) Resolution Number 254 of 2023. https://www.minsalud.gov.co/Normatividad_Nuevo/Resoluci%C3%B3n%20No.%20254%20de%202023.pdf (accessed June 2024).

[41] Department of Health of Southafrica. Regulations relating to the labelling and advertising of foodstuffs. https://www.gov.za/sites/default/files/gcis_document/202304/48428rg11572gon3287.pdf (accessed June 2024).

[42] Taillie LS , Bercholz M , Popkin B et al. (2021) Changes in food purchases after the Chilean policies on food labelling, marketing, and sales in schools: a before and after study. Lancet Planet Health 5, e526–e533. 34390670 10.1016/S2542-5196(21)00172-8 PMC8364623

[43] Taillie LS , Reyes M , Colchero MA et al. (2020) An evaluation of Chile’s Law of Food Labeling and Advertising on sugar-sweetened beverage purchases from 2015 to 2017: a before-and-after study. PLoS Med 17, e1003015. 32045424 10.1371/journal.pmed.1003015 PMC7012389

[44] Dillman Carpentier FR , Correa T , Reyes M et al. (2020) Evaluating the impact of Chile’s marketing regulation of unhealthy foods and beverages: pre-school and adolescent children’s changes in exposure to food advertising on television. Public Health Nutr 23, 747–755. 31822317 10.1017/S1368980019003355 PMC7060093

[45] Snyder LB , Hamilton MA , Mitchell EW et al. (2004) A meta-analysis of the effect of mediated health communication campaigns on behavior change in the United States. J Health Commun 1, 71–96. 10.1080/10810730490271548 14960405

[46] Wakefield MA , Loken B & Hornik RC (2010) Use of mass media campaigns to change health behaviour. Lancet 376, 1261–1271. 20933263 10.1016/S0140-6736(10)60809-4 PMC4248563

[47] Ministry of Health of Chile (2023) Dietary Guidelines for Chile, 3rd ed. Santiago: Ministry of Health of Chile.

[48] Cecchini M , Sassi F , Lauer JA et al. (2010) Tackling of unhealthy diets, physical inactivity, and obesity: health effects and cost-effectiveness. Lancet 376, 1775–1784. 21074255 10.1016/S0140-6736(10)61514-0

[49] Gattrell WT , Hungin AP , Price A et al. (2022) ACCORD guideline for reporting consensus-based methods in biomedical research and clinical practice: a study protocol. Res Integr Peer Rev 7, 3. 35672782 10.1186/s41073-022-00122-0 PMC9171734

[50] Waggoner J , Carline JD & Durning SJ (2016) Is there a consensus on consensus methodology? Descriptions and recommendations for future consensus research. Acad Med 91, 663–668. 26796090 10.1097/ACM.0000000000001092

[51] Vandevijvere S , Barquera S , Caceres G et al. (2019) An 11-country study to benchmark the implementation of recommended nutrition policies by national governments using the Healthy Food Environment Policy Index, 2015–2018. Obes Rev 2, 57–66. 10.1111/obr.12819 30609260

