# Supplementary material for: Potential actions for preventing high consumption of Non-Nutritive Sweeteners among Chilean children and adolescents: recommendations from a panel of relevant actors
Source: Public Health Nutr. 2024 Oct 7;27(1):e199. doi: 10.1017/S1368980024001745 (PMC11505000; doi:10.1017/S1368980024001745)
Supplement: Reyes et al. supplementary material [file S1368980024001745sup001.docx]

SUPPLEMENTARY MATERIAL

*Supplementary Table 1 - Contacted academy experts by topic to evaluate recommendations to prevent excessive Non-Nutritive Sweeteners (NNS) intake in Chilean children and adolescents.*

|  | Investigation topics | Contacted | Agree to participate | Completed  First questionnaire | Completed second questionnaire | Participated in the synchronous session |
| --- | --- | --- | --- | --- | --- | --- |
| Academy experts | Public Policy | 5 | 4 | 2 | 2 | 3 |
|  | Food technology/safety | 4 | 3 | 1 | 1 | 0 |
|  | Microbiota/ Microbiome | 1 | 1 | 0 | 1 | 1 |
|  | Genetics and obesity | 3 | 3 | 3 | 2 | 3 |
|  | Diabetes | 2 | 2 | 2 | 2 | 2 |
|  | Other chronic diseases | 1 | 0 | 0 | 0 | 0 |
|  | Nutrition in infants, children, and adolescents | 5 | 3 | 1 | 2 | 2 |

*Supplementary Table 2 – Contacted organizations by area, recommendations to prevent excessive Non-Nutritive Sweeteners (NNS) intake in Chilean children and adolescents.*

|  |  | Investigation topics | Contacted | Agree to participate | Completed  First questionnaire | Completed second questionnaire | Participated in the synchronous session |
| --- | --- | --- | --- | --- | --- | --- | --- |
| Organizations | Civil organizations | Public Health | 2 | 1 | 1 | 1 | 0 |
|  |  | Popular education in health | 1 | 1 | 1 | 1 | 1 |
|  |  | Nutrition | 1 | 1 | 0 | 1 | 1 |
|  |  | Diabetes | 1 | 1 | 1 | 1 | 1 |
|  |  | Consumers´ associations | 2 | 0 | 0 | 1 | 0 |
|  | Professional organizations | Pediatry | 1 | 1 | 1 | 1 | 0 |
|  |  | Nutrition and obesity | 3 | 3 | 2 | 2 | 2 |
|  |  | Diabetes | 1 | 0 | 0 | 0 | 0 |
|  | Laboratories |  | 6 | 3 | 2 | 2 | 2 |

*Supplementary Figure 1 – Panel conformation for the evaluation of recommendations to prevent excessive Non-Nutritive Sweeteners (NNS) intake in Chilean children and adolescents*

*
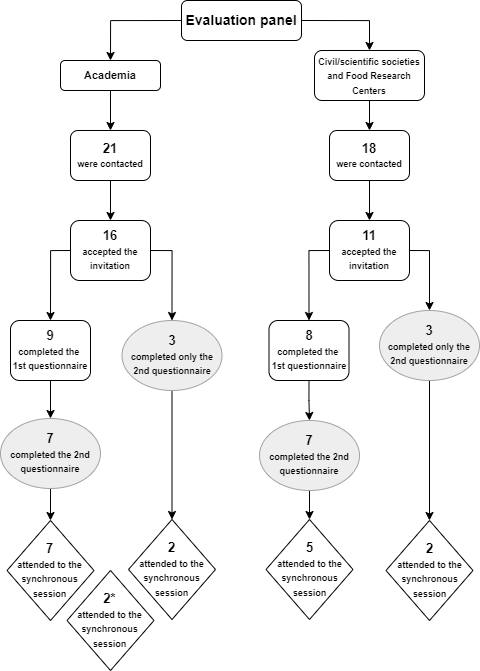
*

** Two workshop attendees who had not participated in any of the previous questionnaires.*

*Supplementary Table 3 - Structure of the first questionnaire to evaluate recommendations to prevent excessive Non-Nutritive Sweeteners (NNS) intake in Chilean children and adolescents.*

| **First Questionnaire** |
| --- |
| **SECTION 1: Initial information** - Documents for the participants to obligatory sign. The next sections of the questionnaire wouldn’t show if the participant didn’t sign the three documents mentioned above or if they didn’t sign the Confidentiality Agreement. |
| - Informed Consent - Confidentiality Agreement - Conflict of Interest |
| **SECTION 2: Participants’ opinions about NNS** – Three open-ended questions to register the experts' overall opinions and insights about NNS consumption in children and adolescents. |
| **Q1.** Do you think NNS consumption helps to prevent childhood obesity? Briefly explain why  **Q2.** Do you think NNS consumption could cause adverse health effects? Briefly explain why  **Q3.** Do you think it's necessary to create and promote actions to prevent excessive NNS consumption in children and adolescents? Briefly explain why |
| **SECTION 3: Recommendation evaluation** – two four-level Likert questions to evaluate the relevance and feasibility of each recommendation. All recommendations included a link to a supplementary document with the background information used to formulate each recommendation. An open-ended section was added for further comments if necessary. The expert panel was asked to evaluate the clarity, pertinence of the language, and availability of information of each recommendation with three dichotomous questions and a free space to write down suggestions. |
| Questions for each recommendation:  **Q1.** Rank the recommendation by RELEVANCE by selecting one of these four options: (1) Non-relevant, (2) Little relevant, (3) Relevant, and (4) Very relevant. Briefly explain why.  **Q2.** Rank the recommendation by FEASIBILITY by selecting one of these four options: (1) Non-feasible, (2) Little feasible, (3) Feasible, and (4) Very feasible. Briefly explain why.  **Q3.** Do you think the language used in this recommendation is pertinent for the target audience? Yes/No  **Q4.** Do you think the recommendation is written clearly and easily understood? Yes/No  **Q5.** Do you think the recommendation includes enough supporting information? Yes/No  **Q6.** If you know more supporting evidence or information about this recommendation, please write it down in this section. If not, please write "no". |
| **SECTION 4: Suggestions and** **comments** - two open questions where the experts could suggest new recommendations that weren’t included or add comments about the subject and the questionnaire. |
| **Q1.** Please write here if you have another recommendation(s) that could be necessary to prevent excessive NNS consumption in children and adolescents.  **Q2.** FINAL COMMENTS: Do you like to add a comment about the recommendations proposed by the research team? |

*Supplementary Table 4 - Structure of the second questionnaire to evaluate recommendations to prevent excessive Non-Nutritive Sweeteners (NNS) intake in Chilean children and adolescents.*

| **Second questionnaire** |
| --- |
| **SECTION 1: Initial information** - The experts that didn’t answer the first questionnaire should sign the Informed Consent, Confidentiality Agreement, and Conflict of Interest Declaration documents. |
| - Informed Consent - Confidentiality Agreement - Conflict of Interest |
| **SECTION 2: First questionnaire results** - General results of the first round were presented, as well as the relevance and feasibility score of all recommendations. |
| **SECTION 3: Re-evaluation** section - the recommendations that didn’t reach any consensus were to rate if they were relevant or feasible, considering the new information from the results of the first round. |
| Questions for each re-evaluable recommendation:  **Q1.** Do you think this recommendation is RELEVANT to prevent excessive consumption of NNS in children and adolescents?  **Q2.** Do you think this recommendation is FEASIBLE to prevent excessive consumption of NNS in children and adolescents? |
| **SECTION 4: Recollection of supplementary information** - in this section, the experts’ panel could add more data about the information provided in the previous round to facilitate its incorporation into the final document. |
| **Q1.** Please write it here if you have more precise information (e.g., references, doi, or links) about the support information. You can also add any additional information. |

*Supplementary Table 5 – Answers to clarity, pertinence, and availability of information questions asked in the first questionnaire to evaluate recommendations to prevent excessive Non-Nutritive Sweeteners (NNS) intake in Chilean children and adolescents.*

|  | Do you think the language used in this recommendation is pertinent for its target population? | | Do you think this recommendation is clear and easy to understand? | | Do you think this recommendation has enough supporting information? | |
| --- | --- | --- | --- | --- | --- | --- |
| 1. To inform the presence of NNS on the front of the package of foods and beverages: *via warning message (i.e., stop sign).* | Yes | No | Yes | No | Yes | No |
|  | 88.24% | 11.76% | 100% | - | 64.71% | 35.29% |
| 2. To inform the presence of NNS on the front of the package of foods and beverages: *via precautionary legend (e.g., as the messages used in Mexico).* | Yes | No | Yes | No | Yes | No |
|  | 88.24% | 11.76% | 94.12% | 5.88% | 64.71% | 35.29% |
| 3. To ban the use of nutritional or health declarations in food and beverages containing NNS. | Yes | No | Yes | No | Yes | No |
|  | 82.35% | 17.65% | 88.24% | 11.76% | 70.59% | 29.41% |
| 4. To ban the sale of food and beverages containing NNS in school environments (e.g., kiosks, school cafeteria, coffee shop, food programs): *only at kindergartens or preschools.* | Yes | No | Yes | No | Yes | No |
|  | 100% | - | 100% | - | 82.35% | 17.65% |
| 5. To ban the sale of food and beverages containing NNS in school environments (e.g., kiosks, school cafeteria, food programs): *only at schools.* | Yes | No | Yes | No | Yes | No |
|  | 100% | - | 100% | - | 76.47% | 23.53% |
| 6. To ban the sale of food and beverages containing NNS in school environments (e.g., kiosks, coffee shops, food programs): *at schools and preschools.* | Yes | No | Yes | No | Yes | No |
|  | 100% | - | 100% | - | 82.35% | 17.65% |
| 7. To implement taxations for food and beverages containing NNS. | Yes | No | Yes | No | Yes | No |
|  | 94.12% | 5.88% | 94.12% | 5.88% | 47.06% | 52.94% |
| 8. To ban special offers (e.g., reduced cost, 2x1) for food and beverages containing NNS. | Yes | No | Yes | No | Yes | No |
|  | 94.12% | 5.88% | 100% | - | 52.94% | 47.06% |
| 9. To restrict marketing directed to children for food and beverages containing NNS. | Yes | No | Yes | No | Yes | No |
|  | 100% | - | 100% | - | 70.59% | 29.41% |
| 10. To regulate NNS content in packaged foods and beverages: *banning the use of NNS.* | Yes | No | Yes | No | Yes | No |
|  | 94.12% | 5.88% | 94.12% | 5.88% | 47.06% | 52.94% |
| 11. To regulate NNS content in packaged foods and beverages: *establishing an allowed NNS maximum concentration.* | Yes | No | Yes | No | Yes | No |
|  | 100% | - | 100% | - | 76.47% | 23.53% |
| 12. To regulate NNS content in packaged foods and beverages: *establishing a maximum number of NNS types allowed to be used in each product.* | Yes | No | Yes | No | Yes | No |
|  | 94.12% | 5.88% | 100% | - | 58.82% | 41.18% |
| 13. To enable and/or strengthen the existing control over the veracity of NNS labeling with a risk-based approach (focus on the main foods/beverages sources of NNS). | Yes | No | Yes | No | Yes | No |
|  | 100% | - | 88.24% | 11,76% | 94.12% | 5.88% |
| 14. To ban the sale of food and beverages containing NNS within 100 meters of schools and preschools. | Yes | No | Yes | No | Yes | No |
|  | 94.12% | 5.88% | 94.12% | 5.88% | 58.82% | 41.18% |
| 15. To ban positioning of foods and beverages containing NNS in privileged shelve areas in stores (i.e., those more visible to children and those in checkout lines). | Yes | No | Yes | No | Yes | No |
|  | 100% | - | 100% | - | 52.94% | 47.06% |
| 16. To promote research and innovation* focused on NNS: monitoring the presence in foods and beverages; identifying technological alternatives for its reduction or replacement; monitoring intake in at-risk populations; studying short-, medium- and long-term health effects. | Yes | No | Yes | No | Yes | No |
|  | 100% | - | 100% | - | 82.35% | 17.65% |
| 17. To implement a health campaign on social media, radio, and television to promote healthy eating and discourage the intake of NNS in children and adolescents. | Yes | No | Yes | No | Yes | No |
|  | 100% | - | 100% | - | 76.47% | 23.53% |
| 18. To add warning messages to discourage the intake of NNS in Food-Based Dietary Guidelines orientated to populations potentially at risk, like pregnant women and children. * | Yes | No | Yes | No | Yes | No |
|  | 94.12% | 5.88% | 94.12% | 5.88% | 76.47% | 23.53% |
| 19. To include (or periodically update, if applicable) warning messages to discourage the intake of NNS in Clinical Guidelines for specific subpopulations such as children under two years old, pediatric population, or pregnant and breastfeeding women. | Yes | No | Yes | No | Yes | No |
|  | 100% | - | 100% | - | 82.35% | 17.65% |
| 20. To include in school curriculum topics such as the health effects of NNS and cooking classes (e.g., decreasing the sweetness of preparations). | Yes | No | Yes | No | Yes | No |
|  | 100% | - | 100% | - | 70.59% | 29.41% |
| 21. To include the potential health effects of NNS in the curriculum of university programs focused on health (Nutrition, Nursery, and Medicine, among others). | Yes | No | Yes | No | Yes | No |
|  | 94.12% | 5.88% | 94.12% | 5.88% | 70.59% | 29.41% |

*Supplementary Table 6 – Emerging categories identified in the content analysis from the first questionnaire to evaluate recommendations to prevent excessive Non-Nutritive Sweeteners (NNS) intake in Chilean children and adolescents.*

| Emerging category | Recommendations that reached consensus to be added to the guide |
| --- | --- |
| Structural changes aimed at the transparency of information | 2. To inform the presence of NNS on the front of the package of foods and beverages: *via precautionary legend (e.g., as the messages used in Mexico).* |
|  | 13. To enable and/or strengthen the existing control over the veracity of NNS labelling with a risk-based approach (focus on the main foods/beverages sources of NNS). |
| Structural changes aimed to prohibit or limit the offer of NNS-containing products | 9. To restrict marketing directed to children for food and beverages containing NNS. |
|  | 15. To ban positioning of foods and beverages containing NNS in privileged shelve areas in stores (i.e., those more visible to children and those in checkout lines). |
| Generation of new evidence about NNS | 16. To promote research and innovation* focused on NNS: monitoring the presence in foods and beverages; identifying technological alternatives for its reduction or replacement; monitoring intake in at-risk populations; studying short-, medium- and long-term health effects. |
| Strength health communication | 17. To implement a health campaign on social media, radio, and television to promote healthy eating and discourage the intake of NNS in children and adolescents. |
|  | 18. To add warning messages to discourage the intake of NNS in Food-Based Dietary Guidelines orientated to populations potentially at risk, like pregnant women and children. |
|  | 19. To include (or periodically update, if applicable) warning messages to discourage the intake of NNS in Clinical Guidelines for specific subpopulations such as children under two years old, pediatric population, or pregnant and breastfeeding women. |
|  | 21. To include the potential health effects of NNS in the curriculum of university programs focused on health (Nutrition, Nursery, and Medicine, among others). |
